# Individualized diagnosis of Parkinson’s disease based on multivariate magnetic resonance imaging radiomics and clinical indexes

**DOI:** 10.3389/fnagi.2025.1504733

**Published:** 2025-01-31

**Authors:** Qianqian Ye, Chenhui Lin, Fangyi Xiao, Tao Jiang, Jialong Hou, Yi Zheng, Jiaxue Xu, Jiani Huang, Keke Chen, Jinlai Cai, Jingjing Qian, Weiwei Quan, Yanyan Chen

**Affiliations:** ^1^Department of Neurology, The First Affiliated Hospital of Wenzhou Medical University, Wenzhou, China; ^2^Department of Cardiology, The First Affiliated Hospital of Wenzhou Medical University, Wenzhou, China

**Keywords:** Parkinson’s disease, MRI radiomics, T1-weighted imaging, T2-FLair, machine learning, clinical-radiomics model

## Abstract

**Objective:**

To explore MRI-based radiomics models, integrating clinical characteristics, for differential diagnosis of Parkinson’s disease (PD) to evaluate their diagnostic performance.

**Methods:**

A total of 256 participants [153 PD, 103 healthy controls (HCs)] from the First Affiliated Hospital of Wenzhou Medical Hospital, were enrolled as the training set, and 120 subjects (74 PD, 46 HCs) from the PPMI dataset served as the test set. Radiomics features were extracted from structural MRI (T1WI and T2-FLair). Support Vector Machine (SVM) classifiers were developed using MRI radiomics data from both monomodal and multimodal radiomics models. The clinical-radiomics model was constructed by integrating clinical variables, including UPDRS, Hoehn-Yahr stage, age, sex, and MMSE scores. Receiver operating characteristic (ROC) curves were generated to evaluate the performance of the models. Decision curve analysis (DCA) was performed to access the clinical usefulness of the models.

**Results:**

In the training set, the T2-FLair and T1WI radiomics model achieved an AUC of 0.896 (95% CI, 0.812–0.900) and 0.899 (95% CI, 0.818–0.908), respectively. The double-sequence radiomics model demonstrated superior diagnostic performance, with an AUC of 0.965 (95% CI, 0.885–0.978) in the training set and an AUC of 0.852 (95% CI, 0.748–0.910) in the test set. The integrated clinical-radiomics model showed enhanced diagnostic accuracy, with AUC = 0.983 (95% CI, 0.897–0.996) in the training set and AUC = 0.837 (95% CI, 0.786–0.902) in the test set. Rad-scores derived from the radiomics model were significantly correlated with diagnostic outcomes (*P* < 0.001). DCA confirmed the substantial clinical usefulness of the clinical-radiomics integrated model.

**Conclusion:**

The integrated clinical-radiomics model offered superior diagnostic performance compared to models based relying solely on imaging or clinical data, underscoring its potential as a non-invasive and effective tool in routine clinical practice for the early diagnosis of PD.

## Introduction

Parkinson’s disease (PD), a debilitating, insidious, and multifaceted neurodegenerative disorder, afflicts over six million individuals worldwide and may experience a twofold increase in prevalence by 2040. Manifestations encompass motor symptoms such as bradykinesia, rigidity, and tremor, alongside nonmotor symptoms, including constipation, hyposmia, sleep disturbances, depression, and cognitive impairment (Kalia and Lang, 2015; Dorsey et al., 2018). Notably, PD ranks second among age-related progressive degenerative conditions, subsequent to Alzheimer’s disease (AD), and is pathologically defined by the presence of α-synuclein-rich Lewy bodies and Lewy neurites (Aarsland et al., 2017; Reich et al., 2022). These pathological changes often precede clinical manifestation by several decades (Bloem et al., 2016).

Currently, the diagnosis is primarily based on the clinical history and motor symptoms (Ben-Shlomo et al., 2024). However, an early-stage disease may be challenging to diagnose due to different clinical manifestations and overlapping symptoms with other neurodegenerative diseases (Heim et al., 2017). Over the past decade, the utilization of imaging as a strategy to diagnosis PD has been significantly highlighted. Imaging phenotypes, beyond pure usage of clinical measures, have the potential to add further value to the assessment of PD (Rahmim et al., 2017; Arnaldi et al., 2017). The pursuit of early and precise PD identification strategies is paramount, as they facilitate prompt therapeutic intervention and ultimately enhance patient survival (Adler et al., 2014). Consequently, identifying imaging markers of PD has been increasingly popular over the years (Acosta-Cabronero et al., 2017; An et al., 2018).

Imaging stands as a cornerstone technology in medicine, facilitating decision-making across screening, diagnosis, therapy, and follow-up endeavors (Aerts et al., 2014; Hood and Friend, 2011). The advent of radiomics in 2012 heralded a novel paradigm shift in image analysis (Avanzo et al., 2020; Lambin et al., 2012). Their quantitative analysis can be used to characterize the biological properties of the imaged volume, such as shape and heterogeneity. This innovative approach harnesses intricate image analysis tools in conjunction with sophisticated statistical methods to uncover the trove of information latent within medical imaging modalities, including computed tomography (CT), magnetic resonance imaging (MRI), and positron emission tomography (PET), which are routinely utilized in clinical practice (Guiot et al., 2022). Brain imaging particularly MRI, has been of key interest in the differential diagnosis of PD (Ohtsuka et al., 2014). As a non-invasive diagnostic tool, MRI is invaluable for diagnosing neurodegenerative diseases and tracking their progression (Adeli et al., 2016). Multimodal MRI for PD diagnosis relies heavily on the detection of subtle structural and functional aberrations within the brain. Researchers have devised and implemented advanced statistical models and machine learning algorithms to analyze quantitative imaging data, thereby facilitating the classification and diagnosis of PD (Adeli et al., 2016; Guiot et al., 2022). The recent years have witnessed unprecedented advancements in data mining, neural networks, deep learning, and other mathematical methodologies, which have been extensively employed in image analysis, exhibiting immense potential in the realm of medical image analysis (Guan et al., 2017). The application of these novel methodologies can enhance the analysis capabilities of intricate multimodal MRI image data, thereby improving the efficacy of PD diagnosis. Emerging evidence underscores the capacity of radiomics to enhance PD diagnosis and clinical assessment, distinguish PD, and forecast prognosis across distinct brain imaging modalities (Sun et al., 2021; Wu et al., 2019; Salmanpour et al., 2021).

However, the integration of radiomic features with clinical data, such as motor and non-motor symptom scores, has been less explored. Combining imaging biomarkers with clinical variables could enhance the sensitivity and specificity of diagnostic models, providing a more comprehensive and robust approach to PD diagnosis. In this study, we aim to develop and evaluate MRI-based radiomics models for differentiating PD from healthy controls (HCs), incorporating both T1-weighted imaging (T1WI) and T2-weighted fluid-attenuated inversion recovery (T2-FLair) MRI sequences. We also integrate clinical features, such as the Unified Parkinson’s Disease Rating Scale (UPDRS), Hoehn-Yahr scale, and cognitive assessment tools, to construct a clinical-radiomics model for improved diagnostic performance. By leveraging multimodal MRI radiomics data and clinical variables, our goal is to provide a non-invasive, reliable diagnostic tool for PD that can be widely implemented in clinical practice.

## Materials and methods

### Design and participants

**FIGURE 1:**
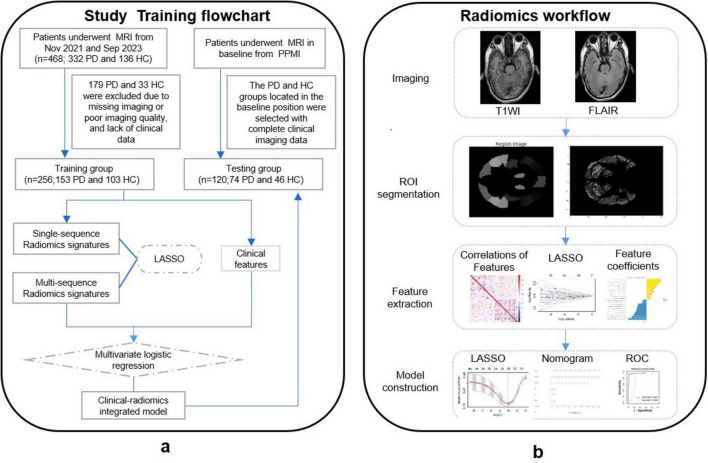
**(A)** Study flowchart. **(B)** Radiomics workflow.

The training set of this study comprised individuals recruited from outpatients and inpatients of the First Hospital of Wenzhou Medical University between November 2021 and September 2023, including 153 participants with PD and 103 participants with HCs. The test set consisted of 120 subjects located at baseline time from participants diagnosed in the Parkinson’s Disease Progression Marker Initiative (PPMI) database^1^ (Adeli et al., 2016), comprising 74 PD and 46 HCs. The study’s workflow is presented in Figure 1.

Ethical approval was granted by the Ethics Committee of the First Hospital of Wenzhou Medical University. Adherence to the principles of the Declaration of Helsinki was paramount, and written informed consent was obtained from all patients prior to the enrollment. The PPMI research program, a global multicenter endeavor, aimed to elucidate the risks and progression of PD through biomarker research. PPMI is an open access data set; data used in the preparation of this manuscript was downloaded from the PPMI database.^2^ The research program and manual are available on this website. Ethical approval for the PPMI study was granted by the review boards of all participating institutions.

### Inclusion and exclusion criteria

Patients with PD included from the First Affiliated Hospital of Wenzhou Medical University and the PPMI database were diagnosed with reference to the most recent diagnostic criteria developed by the Movement Disorder Society (MDS) International. The exclusion criteria for patients with PD were as follows: (1) other Parkinsonian syndromes or secondary etiologies (e.g., multiple system atrophy, progressive supranuclear palsy); (2) a history of stroke, cranial space-occupying lesions, or major neuropsychiatric disorders; (3) previous craniotomy; (4) severe dysfunction of vital organs; (5) poor imaging quality. For comparison, 46 and 103 age- and sex-matched HCs were included in the training and test sets, respectively. The inclusion criteria for the healthy control group were: (1) no significant abnormalities on routine examination; (2) no neuropsychiatric disorders; (3) no family history of PD.

### Clinical neuropsychological evaluation

Motor symptoms in patients with Parkinson’s disease were determined using UPDRS. The Hoehn-Yahr scale determined the stage of PD: grades 1–2.5 for early stage and grades 3–5 for moderately advanced stage. Non-motor symptoms of subjects in the training set were determined by the following scales, such as the Brief Mental Status Examination (MMSE) (Arnaldi et al., 2017), Hamilton Depression Scale (HAMD), Hamilton Anxiety Scale (HAMA), RBD Screening Scale (RBD-SQ), and Ability to Perform Activities of Daily Living (ADL). The non-motor symptoms of the external test set of the PPMI were judged by the following scales: the Montreal Cognitive Assessment Scale (MoCA), Scale of Autonomic Symptoms in Parkinson’s Disease (SCOPA-AUT), Penn State University Smell Identification Test (UPSIT), Epworth Sleepiness Scale (ESS).

### MRI radiomic analysis

#### MRI protocol

MRI data for the training set were acquired at the First Hospital of Wenzhou Medical University using either a 1.5T or 3.0T superconducting MRI scanners. The scans encompassed T2-Flair and T1WI sequences, with the scanning baseline aligned parallel to the anterior-posterior commissure and encompassing a continuous range from the parietal lobe apex to the inferior cerebellar hemisphere. In test set, the imaging features, derived from PPMI dataset, were acquired as T2-Flair and T1WI sequences on a Siemens MRI scanner (MAGNETOM Trio Tim, Siemens Healthcare, Germany) operating at either 1.5T or 3.0T. The PPMI neuroimage database was populated until October 2023, adhering to strict acquisition protocols, including a layer thickness of 1.5 mm or less and zero interscan gaps. Repetition times and echo times were tailored to the recommended settings for each scan type. Comprehensive details regarding the MRI scanning sequences, specific imaging parameters, and methodologies were outlined in the PPMI Magnetic Resonance Imaging Operator’s Manual, providing a definitive guide to the equipment, parameters, and imaging practices employed within the PPMI project.

#### Image acquisition and reconstruction of MRI sequences

In this study, MRI images acquired from the First Hospital of Wenzhou Medical University and PPMI database were converted from DICOM format to NIfTI format using the dcm2niix tool in the MRIcroGL software, preserving the DICOM metadata in the images during the conversion process. Preprocessing steps were performed using Matlab 2021b and SPM 12 software packages, including head motion correction, alignment, segmentation, normalization and smoothing. Head-motion correction was performed using SPM’s Realign function, using rigid alignment and optimized to least squares. Structural images are aligned to the MNI standard space by the Coregister function, using affine alignment and cubic spline interpolation. The segmentation step used the Segment function to segment the structural image into gray matter, white matter, and cerebrospinal fluid, choosing a Gaussian mixture model for the segmentation. Normalization uses a nonlinear approach to normalize the data to MNI space with an elastic deformation model to accommodate individual differences. Finally, the data were smoothed by the Smooth function of SPM using a 6–12 mm isotropic smoothing kernel to improve the signal-to-noise ratio. All preprocessing steps were performed according to SPM default settings or adjusted according to data quality, ultimately generating high-quality brain imaging data that can be used for subsequent statistical analysis.

#### Region-of-interest segmentation, image preprocessing and feature extraction

We utilized the Automated Anatomical Labelling Atlas (AAL3v1) as the baseline brain template for the ROI extraction work using Python 3.9 and the nibabel toolkit under the Anaconda 3 platform. The AAL3v1 template is a version of the AAL3 template designed to provide more fine-grained and efficient labeling of anatomical regions. Similar to the AAL3 template, the AAL3v1 template was used for automated segmentation and region labeling of brain images by dividing the brain into multiple standardized anatomical regions (Huppertz et al., 2016). It accurately identified each anatomical region of the brain by aligning individual brain images with the AAL3v1 standardized space. Specifically, we loaded the AAL 3v1 standard brain partition atlas and extracted specific brain ROIs from it, including the caudate nucleus (AAL: 75, 76), the nucleus accumbens (AAL: 77, 78), the pallidum (AAL: 79, 80), the red nucleus (AAL: 45, 46), the substantia nigra (AAL: 161, 162, 163, 164, 165, 166), frontal (AAL: 3-20), parietal (AAL: 63–66), occipital (AAL: 53–58), and temporal (AAL: 85–94) lobes. The extraction results of these brain regions were saved as NIFFTI files as ROIs for this study.

We adopted an AI-based machine learning approach using Python version 3.9 under the Anaconda 3 platform and applied the feature extractor component of the pyradiomics package to extract features from image histology data in the region of interest. The calculation method for each histological feature can be found in the open source pyradiomics v3.0.1 package description. The pyradiomics toolkit was used to extract image histological features from all ROIs labeled in T2-Flair, T1WI images in training and test sets, respectively. These extracted features included different types of features: first-order features, second-order features and higher-order features. Finally, 1074 imaging histological features were extracted from each ROI in the T2-Flair and T1WI images of each subject.

Statistical analyses and feature screening were performed using R language packages (e.g., dplyr, ggplot2, caret). An independent samples t-test was utilized to identify differing histological features. Features with *P* > 0.05 were excluded, while those with *P* < 0.05 were retained. Following initial screening, the Least Absolute Shrinkage and Selection Operator (LASSO) was applied to further refine the significant imaging histological features. LASSO is a regularized machine learning algorithm, suitable for performing dimensionality reduction of high-dimensional data. This approach minimized the coefficients of non-significant features unrelated to classification and diagnosis to zero, thereby reducing the influence of irrelevant features on the model and enhancing interpretability. The optimal feature subset was constructed from the training set of imaging genomics features using ten-fold cross-validation. The optimal λ-value was determined by calculating the Mean Squared Error (MSE) for different values of λ. For each λ, the model’s performance was evaluated using cross-validation, and the λ that yielded the lowest average MSE across all folds was selected as the optimal regularization parameter. The feature weight coefficients were derived from the corresponding coefficients list. Using the optimal λ-value, we constructed the optimal feature subset containing the selected histological feature variables and applied it to the entire training set.

#### Development and evaluation of radiomics models

A Support Vector Machine (SVM) classifier model was trained using the filtered optimal feature subsets derived from T2-Flair and T1WI in training datasets, respectively. The model was subsequently applied to the test set for classification purposes. Receiver operating characteristic (ROC) curves were generated, and performance metrics, including the area under the curve (AUC), accuracy, sensitivity, specificity, and predictive values, were computed.

The T2-Flair and T1WI radiomics features in training sets were filtered using a feature selection approach to obtain smaller, more manageable feature subsets. These subsets were then merged to create a newly comprehensive training set. Using this merged training set and the corresponding target variables, a LASSO regression model was applied, and ten-fold cross-validation was employed to identify the best feature subset. Ten-fold cross-validation can effectively assess the robustness and generalization ability of the model by training and validating the model multiple times, avoiding the assessment bias caused by data division chance. The feature indices corresponding to non-zero coefficients in the LASSO regression model were selected as the optimal feature subset. Subsequently, the SVM classifier model was utilized to classify the corresponding test set data based on this double sequential optimal feature subset. ROC curves were plotted for the SVM classifier’s classification in both training and test sets, and AUC values were calculated yet.

Furthermore, in addition to generating ROC curves and AUC values to evaluate the model’s diagnostic performance, features with non-zero coefficients were selected for use in the modeling and training process using the LASSO regression model and SVM classification algorithms. This selection process included considerations of accuracy, 95% confidence intervals, sensitivity, specificity, positive predictive value and negative predictive value. Finally, imaging histology scores (rad-score and nomo-score) were calculated for each subject by multiplying the selected features by their respective correlation coefficients.

#### Clinical-radiomics-laboratory diagnostic model construction

Initially, the assessment of clinical variables pertaining to non-motor symptoms revealed differing scales between the training set and test set. To address this, we referenced a study detailing an MMSE and MOCA conversion table (Bergeron et al., 2017). Based on the findings of this reference, we converted MOCA to MMSE values using the provided conversion table. Consequently, all subsequent references to MOCA values from the external test set within this study were adjusted to MMSE values to ensure consistency in further analyses. The same clinical variables for PD and HCs, such as age, years of education, UPDRS, UPDRS I-IV, and Hoehn-Yahr were collected and compared in the training and test sets. And the clinical feature models were constructed by logistic regression models and SVM classifier models. The clinical-radiomics model was built by multivariate logistic regression analysis with a ten-fold cross-validation and SVM classification. ROC curves were performed to evaluate the diagnostic performance of the constructed model to distinguish PD from HCs. A nomogram was established based on clinical-radiomics model. The calibration of nomogram was analyzed by the Hosmer–Lemeshow test and calibration curves. To assess the clinical utility of these models, decision curve analysis (DCA) was calculated.

### Statistical analysis

Most statistical analyses were performed on R software (v4.0.2)^3^ and Python 3.9. Also, Shapiro-Wilk test, Pearson chi-square test, independent samples t-test, and Wilcoxon rank sum test in SPSS software were used to analyze the within-group differences in the clinical characteristics. Continuous variables were expressed as mean ( ± standard deviation) or median (interquartile spacing), and categorical variables were expressed as frequency (percentage). The independent sample *t*-test was performed to analyze normally distributed continuous variables. The Wilcoxon rank sum test was employed to analyze the continuous variable with abnormal distribution. Correlations between screened imaging histologic features and clinical characteristics were then assessed using Spearman’s rank correlation analysis. The mRMR algorithm, LASSO logistic regression, and ROC curves were executed by the “mRMRe,” “Glmnet,” and “pROC” packages, respectively. AUCs of these models were compared using the DeLong test (Demler et al., 2012). The power analysis was performed by using the pwr.t2n.test, and empirical effect size was also calculated. A two-tailed *P*-value < 0.05 was considered statistically significant.

## Results

### Demographic characteristics and clinical assessment

This analysis encompassed 256 participants recruited from November 2021 to September 2023 at The First Affiliated Hospital of Wenzhou Medical University as the training set. This comprised 153 patients with PD and 103 HCs. Additionally, 120 subjects (74 PD and 46 HCs) from the PPMI dataset constituted the test set. In the training set, the mean age at evaluation was 66.83 ± 7.68 years for PD patients and 65.10 ± 7.61 years for HCs (*P* > 0.05). Similarly, in the test set, the mean age was 65.25 ± 7.28 years for PD patients and 62.44 ± 7.43 years for HCs (*P* > 0.05). Table 1 outlines the demographic characteristics and clinical assessments of all participants. Baseline comparisons revealed no significant differences in age, sex distribution, or educational level between PD and HC groups in both the training and test sets. In the training set, PD patients exhibited significantly higher scores on motor (UPDRS, Hoehn-Yahr, ADL) and non-motor (MMSE, HAMD, HAMA) symptom scales (*P* < 0.01) compared to HCs. In the test set, significant differences were observed for Hoehn-Yahr, UPDRS, MoCA, UPSIT, ESS, and SCOPA-AUT (*P* < 0.01). However, there was no significant difference in the MMSE scores after MOCA conversion between the PD and HCs in test set.

**TABLE 1:** Subject demographic information tables for the training and test sets.

	Training set				Test set		
	**PD**	**HCs**	***P-*value**		**PD**	**HCs**	***P-*value**
Age ^a^	66.83 ± 7.68	65.10 ± 7.61	0.081	Age ^a^	65.25 ± 7.28	62.44 ± 7.43	0.051
Sex(F%) ^c^	64(41.8%)	56(54.4%)	0.112	Sex(F%) ^c^	31(43.8%)	18(39%)	0.299
Years	4[1,6]	/	/	Years	4[2,7]	/	/
Education(year)^b^	3[0,6]	5[0,8]	0.115	Education(year)^b^	16[14,18]	16[14,18]	0.473
UPDRS ^b^	49[31,62.5]	1[0,2]	<0.001	UPDRS ^b^	32[26,48]	5[3,10]	< 0.001
I	1[1,3]	0[0,1]	<0.001	I	5[3,6]	2[1.25,5.75]	0.108
II	13[8,18]	0[0,0]	<0.001	II	7[4,10]	0[0,0]	<0.001
III	29[18,41.75]	0[0,0.5]	<0.001	III	22[16,35]	1[0,3]	<0.001
IV	2[0,4]	0[0,1]	<0.001	IV	2[0,4]	0[0,1]	<0.001
Hoehn-Yahr ^b^	3[2,4]	0[0,0]	<0.001	Hoehn-Yahr ^b^	2[1,2]	0[0,0]	<0.001
MMSE ^b^	20[14,24]	24[20,27]	<0.001	MOCA ^b^	27[26,29]	28[27,29]	0.073
HAMD ^b^	5[2,9]	3[1.5]	<0.001	SCOPAAUT ^b^	27[20,29]	23[14.29]	<0.001
HAMA ^b^	8[4,12.5]	4[2,7]	<0.001	UPSIT ^b^	23[16,28]	32[29,36]	<0.001
RBD ^b^	13[2,26.5]	3[1,9]	<0.001	ESS ^b^	5[2,7]	4[4,7]	<0.001
ADL ^b^	31[22,41]	20[20,20]	<0.001	MOCA-MMSE ^b^	28[27,30]	29[28,30]	0.769

*^a^*Represents the measurement data conforming to the normal distribution, the results are expressed as the mean (standard deviation), the one-way ANOVA.

*^b^*Represents the measurement data, the median value [25th percentage, 75th percentage], the Wilcoxon rank sum test.

*^c^*Represents the count data as the number of individuals (percentage), the χ^2^-test. All pairwise comparisons were performed using Bonferroni-corrected post-hoc tests.

### Feature selection in MRI radiomics

**FIGURE 2:**
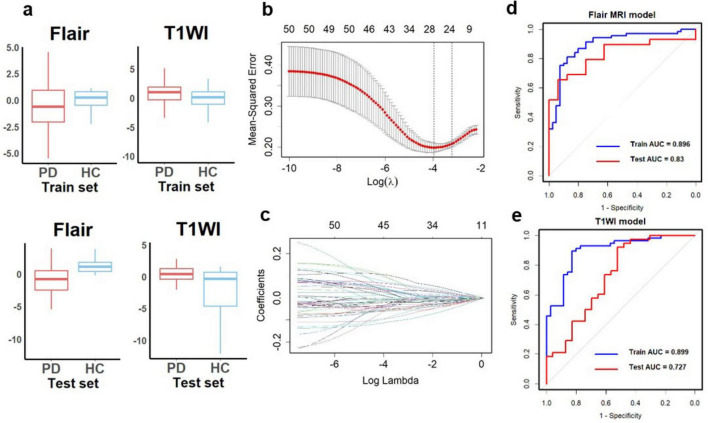
Diagnostic performance of radiomics signatures in single-sequence models. **(A)** The radiomics signatures of two sequences showed significant differences between PD and HCs (*P* < 0.05) in the training and test sets. **(B**) Texture reduction and selection used LASSO based on the minimum 1 with 10-fold cross-validation. The Y-axis indicates binomial deviances, and the X-axis means the number of features. **(C)** The value of l with the number of features. The ROC curves of single-sequence models in **(D)** Flair MRI and **(E)** T1WI. LASSO, least absolute shrinkage and selection operator; ROC, receiver operating characteristic; AUC, the area under the curve.

A flowchart of image processing and analysis is presented in Figure 1. The MRI-based feature selection was used to decrease the redundancy of the feature set and build an optimal subset of complementary features. A total of 1,074 radiomic features were initially extracted from each ROI within the T2-Flair and T1WI MRI images across the 10 predefined ROIs in the training set. Feature selection was performed using the t-test or Wilcoxon rank-sum test, which led to the retention of 716 features from T2-Flair and 1644 features from T1WI images. All the radiomics signatures showed significant differences between PD and HCs (*P* < 0.05). Details on these features and dimensionality reduction are provided in Figure 2A. Subsequently, the feature set yielding the highest mean accuracy in the test set was selected as the optimal feature subset. After feature dimension reduction, the top 20 features were identified as the optimal subset for further analysis, as shown in Figure 2B. These 20 features were then used to construct an optimized radiomics model based on both T2-FLair and T1WI sequences. The final selection of the top 20 radiomics features was refined using LASSO regression, as shown in Table 2 and Figure 2C.

**TABLE 2:** Sking of feature weight coefficients after LASSO screening (top 10 displayed).

MRI	ROI	Features	Coefficient
**Flair**			
	Red_NL	waveletLLHgldmLargeDependenceLowGrayLevelEmphasis	−0.07564
	Pallidum_R	waveletHHLgldmZoneVariance	0.05895
	Red_NL	waveletHLHgldmLargeDependenceHighGrayLevelEmphasis	−0.05505
	Frontal_Inf_Orb_2R	waveletLHHglszmLargeAreaLowGrayLevelEmphasis	0.05342
	SN_pr_L	waveletHHLglszmLargeAreaHighGrayLevelEmphasis	0.04792
	Putamen_R	waveletLHHglszmZoneVariance	−0.04635
	Frontal_Inf_Orb_2R	waveletHLLglszmLargeAreaHighGrayLevelEmphasis	−0.04154
	Pallidum_L	waveletHLLgldmLargeDependenceHighGrayLevelEmphasis	0.03803
	Red_NR	waveletLHLgldmLargeDependenceHighGrayLevelEmphasis	−0.03747
	Pallidum_L	waveletHLHgldmLargeDependenceLowGrayLevelEmphasis	−0.03535
**T1WI**			
	SN_pr_R	waveletHLHglszmZoneVariance	−0.09301
	Pallidum_R	waveletLLHfirstorder90Percentile	0.06475
	Frontal_Inf_Oper_R	waveletHHHgldmLargeDependenceEmphasis	−0.06310
	Occipital_Sup_L	waveletLHHfirstorder90Percentile	−0.05769
	Red_NL	waveletHLHglszmZoneVariance	0.05030
	Temporal_Mid_L	waveletLLLfirstorderMinimum	−0.05017
	SN_pc_L	waveletHLLfirstorder90Percentile	−0.04523
	SN_pc_R	waveletLLHfirstorderMaximum	−0.04481
	Temporal_Inf_R	waveletHLHfirstorderKurtosis	−0.04051
	SN_pc_R	waveletLLHfirstorderMean	−0.04005

**FIGURE 3:**
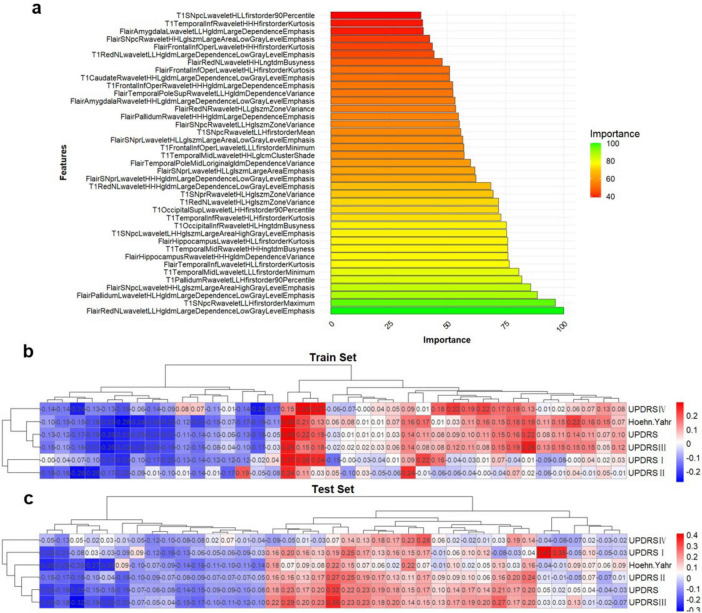
Multi-sequence feature selection. **(A)** Top 20 features after multi-sequence screening. **(B,C)** Correlation between imaging omics and clinical features in the training and test sets. *Represents a *P*-value of < 0.05.

In addition, the filtered features were ranked by their importance, and correlation analyses were performed between each feature in both training and test sets (Figure 3A). This study had also conducted an association analysis of the extracted final features of the ROIs and the clinical features in the dataset (Figures 3B,C). Specifically, all brain region features were derived from higher-order imaging omics features after wavelet transform treatment, while 7 features are from SN (6 features from SNpc, 1 feature from SNpr), 4 features from Pallidum, 4 features from Red nucleus, 3 features from Frontal lobe, 1 feature from Putamen and 1 feature from Occipital lobe. Notably, features reflecting SN are most important for the diagnosis of PD, which also validates the pathogenesis of PD. In addition, wavelet HHL glszm Large Area High Gray Level Emphasis in the SN _ pc_ L brain region in Flair MRI was correlated with UPDRS I, UPDRS II, UPDRS III, UPDRS III and Hoehn-Yahr (Figure 3C). This suggests that wavelet-based texture features derived from the substantia nigra are closely linked to both the clinical severity and the progression of PD.

### Performance of radiomics features

Using the optimal feature subset derived from T2-FLair and T1WI MRI images, a SVM classifier was developed and evaluated. The classifier’s performance was assessed on the training dataset by comparing two monomodal radiomics models, one based on T1WI images and the other on T2-Flair images. ROC analysis was employed to evaluate the diagnostic efficiency of these MRI models (Figures 2D,E). The T2-Flair MRI radiomics model achieved an AUC of 0.896 in the training set, with a sensitivity of 75.0% and a specificity of 91.3% (Table 3). The T1WI MRI radiomics model also demonstrated high diagnostic accuracy, with an AUC of 0.899, a sensitivity of 74.2%, and a specificity of 92.9% in the training set (Table 3). The DeLong test indicated no statistically significant difference between the two monomodal MRI radiomics models (*P* = 0.738 for T1WI vs. T2-Flair), suggesting comparable performance.

**TABLE 3:** Diagnostic performance of imaging omics features in T2-Flair and T1WI models.

	T2-Flair MRI		T1WI MRI	
	**Training set**	**Test set**	**Training set**	**Test set**
AUC	0.896	0.830	0.899	0.727
Accuracy	0.853	0.755	0.859	0.738
**95% CI**				
Lower	0.812	0.742	0.818	0.645
Upper	0.900	0.854	0.908	0.797
Sensitivity	0.750	0.649	0.742	0.621
Specificity	0.913	0.896	0.929	0.868
PPV	0.823	0.817	0.830	0.754
NPV	0.784	0.746	0.735	0.648

AUC, Area under the ROC curve; Accuracy, Accuracy; 95% CI, 95% confidence interval; Lower Power (Lower limit), lower limit of confidence interval; Upper (Upper limit), upper limit of confidence interval; PPV, positive predictive value; NPV: negative predictive value.

**FIGURE 4:**
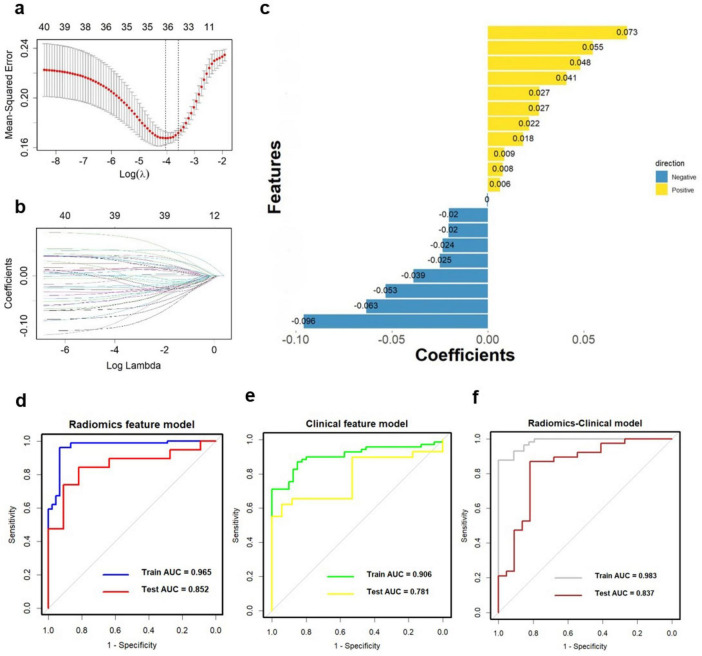
Multiple-sequence feature selection and radiomics-based classifier construction. **(A,B)** LASSO. **(C)** Coefficients of features in Radiomics model. The ROC curves of the Radiomics model (**D**, AUC = 0.965), clinical feature model (**E**, AUC = 0.906), and Radiomics-clinical model (**F**, AUC = 0.983). ROC, receiver operating characteristic; AUC, the area under the curve; LASSO, least absolute shrinkage, and selection operator.

To assess the discriminative ability of the radiomics signatures, rad-score and nomo-score were calculated in both the training and test sets (Table 4). The rad-score showed a significant correlation with diagnostic performance in distinguishing PD from HCs in both the training and test sets (*P* < 0.001). However, for the differential diagnosis between PD and HCs, there were no statistically significant differences in the nomo-score of single-sequence models in either training or test set (*P* > 0.05). Thus, categorizing PD patients from HCs can be effectively achieved based on the rad-score derived from MRI radiomics signatures. After harmonizing the radiomics features, a double-sequence imaging omics was obtained using LASSO regression (Figures 4A–C). Based on the optimal double-sequence radiomics model, the SVM classifier was applied to provide classification information using MRI radiomics signatures in the test set. The diagnostic performance of the radiomics models was assessed through ROC curve analysis in both the training and test sets (Figure 4D). The reconstructed radiomics model exhibited excellent diagnostic accuracy, with an AUC of 0.965 (95% CI, 0.885–0.978) in the training set and an AUC of 0.852 (95% CI, 0.748–0.910) in the test set (Table 5; Figure 4D).

**TABLE 4:** Results of rad-score and nomo-score in the T2-Flair and T1WI imaging omics models

	T2-Flair MRI						T1WI MRI					
	**Training set**			**Test set**			**Training set**			**Test set**		
	**PD**	**HC**	***P-*value**	**PD**	**HC**	***P-*value**	**PD**	**HC**	***P-*value**	**PD**	**HC**	***P-*value**
Rad-score	0.897	0.704	<0.001	0.899	0.775	<0.001	0.904	0.797	<0.001	0.723	0.64	<0.001
Nomo-score	0.141	0.289	0.069	0.128	0.589	0.051	0.095	0.187	0.245	0.357	0.449	0.157

Rad-score and nomo-score are both two imaging-omics assessment tools.

**TABLE 5:** Diagnostic performance of imaging omics features in the combined model.

	Radiomics model		Clinical feature model		Clinical-radiomics model	
	**Training set**	**Test set**	**Training set**	**Test set**	**Training set**	**Test set**
AUC	0.965	0.852	0.906	0.781	0.983	0.837
Accuracy	0.942	0.800	0.835	0.739	0.923	0.783
**95% CI**						
Lower	0.885	0.748	0.758	0.726	0.897	0.786
Upper	0.978	0.910	0.917	0.869	0.996	0.902
Sensitivity	0.911	0.636	0.875	0.882	0.912	0.790
Specificity	0.960	0.737	0.811	0.655	0.930	0.894
PPV	0.939	0.743	0.841	0.817	0.968	0.782
NPV	0.926	0.775	0.763	0.669	0.955	0.813

AUC, Area under the ROC curve; Accuracy, Accuracy; 95% CI, 95% confidence interval; Lower Power (Lower limit), lower limit of confidence interval; Upper (Upper limit), upper limit of confidence interval; PPV, positive predictive value; NPV, negative predictive value.

### Construction and evaluation of clinical-radiomics integrated model

In combination with clinical variables, we further constructed an integrated diagnosis model by logistic regression. Multivariable logistic regression analysis was performed to select independent predictors for PD, including demographic and clinical indicators. The UPDRS, UPDRS I, UPDRS II, UPDRS III, UPDRS IV and Hoehn-Yahr were identified as independent factors in the clinical-radiomics integrated model. We also evaluated the diagnostic efficiency of the clinical model using ROC analyses. The clinical model yielded an AUC of 0.906 (95% CI, 0.758-0.917) in training set (Table 5; Figure 4E), and 0.781 (95% CI, 0.726-0.869) in test set. Finally, A more optimized clinical-radiomics integrated model was developed with ten-fold cross-validation and SVM classifier to distinguish PD from HCs. We also conducted the discriminatory efficiency of the clinical-radiomics integrated model using ROC analyses. The clinical-radiomics model yielded AUC of 0.983 (95% CI, 0.897–0.996) in training set (Table 5; Figure 4F), and 0.837 (95% CI, 0.786–0.902) in test set, which was statistically different from the radiomics model by the DeLong test (*P* < 0.05).

**FIGURE 5:**
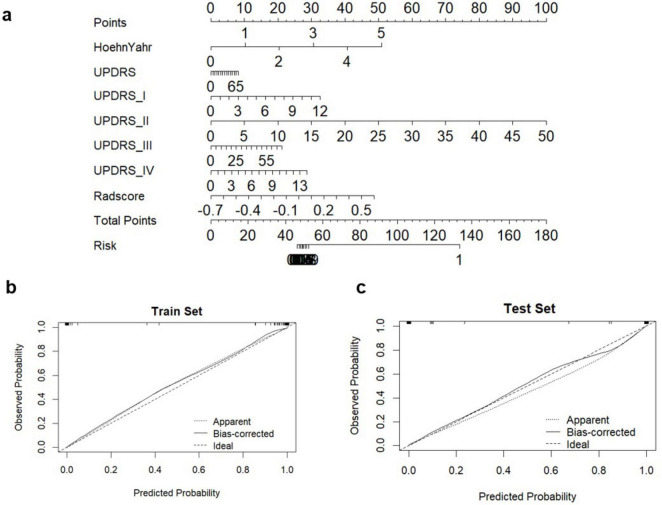
Diagnostic evaluation and test of clinical-radiomics integrated models for differentiation of PD and HCs. **(A)** A nomogram based on clinical characteristics and Rad-score. **(B,C)** Calibration curves for clinical-radiomics integrated model in train set and test set.

**FIGURE 6:**
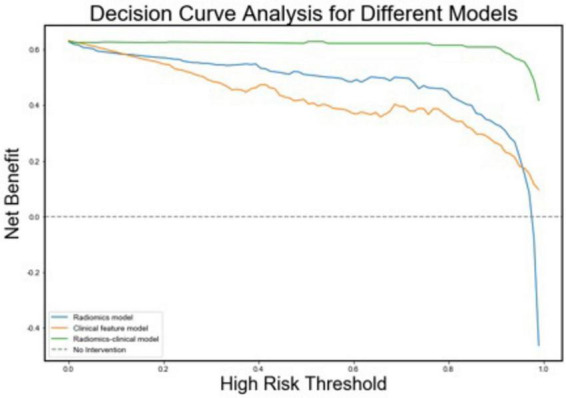
Decision curves for the radiomics, clinical, and radiomics-clinical integrated models. The Y-axis shows the model benefit. The green line represents the radiomics-clinical integrated model. The blue line represents the radiomics model, and the yellow line represents the Clinical model. The X-axis means the threshold probability.

Additionally, we investigated and compared rad score and nomo score of the double-sequence imaging omics and clinical-radiomics integrated model (Table 6). In both training and test sets, rad score showed statistical differences (*P* < 0.001) in the multimodal radiomics model and clinical-radiomics integrated model. There was significantly different in nomo-score in clinical-radiomics model in both train and test sets (*P* < 0.001). Nomo-score of the double-sequence imaging omics was only different in test set (*P* = 0.05). But no significant difference in training set was observed in nomo-score of the multimodal radiomics model (*P* > 0.05). The multimodal radiomics model and clinical-radiomics integrated model compared to single-sequence imaging appeared to have higher diagnostic efficacy. The nomogram based on clinical factors and Rad-score was shown in Figure 5A. The performance and reliability of the model was evaluated by the calibration curve and the Hosmer–Lemeshow test (Figures 5B,C). The Hosmer–Lemeshow test in the radiomics-clinical integrated model showed no significant differences in the goodness-of-fit for the training set (*P* = 0.999). Subsequently, DCA was conducted to evaluate clinical utility of the clinical model, radiomics models, and clinical-radiomics integrated model in the training and test sets. The result of DCA demonstrated that the benefit of clinical-radiomics integrated model was relatively higher compared other models to differentiate PD from HCs in a range from 0 to 1, as shown in Figure 6. Considering the relatively small sample size, effect size and confidence interval were calculated to measure the practical significance. The result showed a 0.8 effect size was detected with 98% confidence, a maximum of 4.78% probability of misreporting differences. Moreover, the value of empirical effect size was calculated (*d* = 2.071). The result showed that the effect size between the two groups was large and the difference in the mean between the two groups was significant, which indicated that the empirical sample size included in this study could support the conclusion.

**TABLE 6:** Results of rad-score and nomo-score in the combined model.

	Radiomics model						Clinical-radiomics model					
	**training set**			**test set**			**training set**			**test set**		
	**PD**	**HC**	***P-*value**	**PD**	**HC**	***P-*value**	**PD**	**HC**	***P-*value**	**PD**	**HC**	***P-*value**
Rad-score	0.958	0.793	<0.001	0.897	0.732	<0.001	0.983	0.897	<0.001	0.825	0.784	<0.001
Nomo-score	0.047	0.193	0.092	0.138	0.173	0.050	0.046	0.006	<0.001	0.268	0.122	<0.001

Rad-score and Nomo-score are both two imaging-omics assessment tools

## Discussion

Given accurate diagnosis of PD remains challenging, particularly in early stage (Peralta et al., 2019). The disease-modifying therapies might be ineffective to hinder the neuro-degeneration progression. There is a calling need of automated approaches and techniques as prior tools to detect brain alterations in PD. Using a multivariate approach, we evaluated the diagnostic performance of radiomics features extracted from T2-Flair and T1WI MRI sequences, and constructed an integrated clinical-radiomics model to improve PD detection accuracy. We successfully developed an optimal radiomics signature and clinical feature constructed model for detection of PD by most frequently used imaging methods in clinics, with an excellent performance. These findings highlight that the approach to identify PD by integrating MRI radiomics and clinical characteristics could be potentially feasible in clinics.

The demographic characteristics of the participants, including age and sex distribution, did not differ significantly between PD and HCs in either the training or test set, suggesting that our groups were well-matched for these baseline factors. This is crucial to ensure that the observed diagnostic performance reflects the model’s capacity to identify disease-specific characteristics, rather than being influenced by variations in demographic factors.

Segmentation in image analysis remains challenging due to the complicated morphology of the brain. The methods of brain structural segmentation contain manual and automatic segmentation (Ewert et al., 2019). Currently, manual segmentation is frequently used in clinics because of its more accurate results than automatic segmentation (Hu et al., 2021; Ewert et al., 2019). However, manual segmentation is not only time-consuming but also necessitates extensive anatomical knowledge from the operator (Zwirner et al., 2017; Chakravarty et al., 2013). Thus, this study focused on automatic segmentation, which relied on algorithmic methods. Initially, we performed precise co-registration of frequently used MRI images, including T1WI and T2-Flair. The images were subsequently segmented and normalized into standard AAL3v1 space using the unified segmentation approach and tissue probability maps. As a result, the use of automatic segmentation methods often ensures reproducibility and objectivity in the outcomes.

The aim of this study was to evaluate the potential of MRI radiomics in distinguishing PD from HCs. MRI radiomics, which involves the extraction of high-dimensional quantitative features from medical images, has gained considerable attention due to its ability to capture subtle changes in clinics (Xu and Zhang, 2019). The MRI radiomics analysis involved selecting features from both T2-Flair and T1WI MRI images. Using statistical tests such as t-tests and Wilcoxon rank-sum tests, we identified a total of 716 features from T2-Flair and 1644 features from T1WI, all of which showed significant differences between PD and HC groups. The dimensionality reduction process, through the application of LASSO regression, allowed for the identification of the top 20 radiomic features that provided optimal diagnostic performance. The significant differences in these radiomics signatures between PD and HCs (*P* < 0.05) suggested a distinctive imaging pattern in PD that could be effectively captured through advanced machine-learning algorithms, in consistent with previous studies (Sun et al., 2024). In the constructed radiomics model, the optimal features were primarily distributed in the substantia nigra, frontal lobe, temporal lobe, occipital lobe, hippocampus, and globus pallidus. Notably, the most prominent features were derived from the SN, including both the SNpc and SNpr, which are key regions implicated in PD pathology due to the degeneration of dopaminergic neurons (Guiot et al., 2022). For patients with PD, widespread functional abnormalities are indeed observed in the temporal lobe, frontal lobe, and hippocampus (Choi et al., 2016). Additionally, features extracted from other brain regions such as the pallidum, red nucleus, frontal lobe, putamen and occipital lobe further supported the role of basal ganglia and motor related brain structures in PD pathophysiology.

The radiomics model developed in this study predominantly relies on features derived from wavelet transform, capturing image details across multiple scales. By decomposing images into high-frequency components (representing fine details and edges) and low-frequency components (reflecting large-scale structures), wavelet transform provided a more detailed description of texture features. This approach is particularly effective for detecting subtle pathological changes, such as microstructural alterations in the substantia nigra (SN), which are hallmark features of PD (Betrouni et al., 2021). Our findings highlight the diagnostic advantages of wavelet-derived features, which enhance the model’s accuracy, sensitivity, and specificity for PD. Interestingly, the wavelet-based feature “Large Area High Gray Level Emphasis” from the SN pars reticulata (SNpr) showed a strong correlation with motor function as measured by UPDRS scores. This underscores the importance of SN-related features in reflecting the motor deficits characteristic of PD, consistent with the pathophysiological model of PD as a disorder primarily involving the basal ganglia circuitry (Mitchell et al., 2021). These features, which reflect the complexity and heterogeneity of brain tissue, may capture subtle variations in neuronal degeneration and neuroinflammation, which are characteristic of early-stage PD. The association between radiomics features and clinical measures reinforces the potential of using radiomics as a non-invasive biomarker for PD identification (Mitchell et al., 2021).

In the radiomics analysis, we first demonstrated the diagnostic performance of the monomodal radiomics models of T1WI and T2-Flair separately. The top-performing radiomics models based on T2-FLair and T1WI sequences showed comparable diagnostic accuracy, with AUC values of 0.896 and 0.899, respectively, in the training set. The integration of both imaging sequences into a monomodal radiomics model yielded a superior AUC of 0.965, highlighting the utility of combining multimodal imaging data to enhance diagnostic performance. This aligns with findings from other studies that have used multimodal MRI approaches to distinguish PD from other neurodegenerative conditions (Sun et al., 2024). However, structural changes often precede these clinical manifestations, and may be detectable through advanced imaging techniques. Subtle changes that cannot be observed regularly by the naked eye can be reflected by radiomics that provides a highly sensitive opportunity to estimate the distribution of structural information at the microscopic level (Ruan et al., 2020). Studies have shown that these histological changes can be quantified using T2-Flair and T1WI MRI sequences, with radiomic features capturing the alterations in texture, intensity, and heterogeneity of these brain regions (Sun et al., 2024; Kang et al., 2022). The synergistic combination of T1WI and T2-Flair may enhance the model’s ability to provide a more comprehensive representation of the underlying pathology. While T1WI primarily offers detailed anatomical insights, T2-Flair is highly sensitive to tissue property changes, which are often critical for distinguishing between tissue types or detecting subtle lesions (Tuite, 2016). By integrating these modalities, the multimodal radiomics model harnesses the complementary strengths of both sequences, thereby enhancing diagnostic accuracy, despite the lack of statistically significant differences observed between the two monomodal radiomics models. The ability of multimodal radiomics model to detect these early, subtle changes offers an advantage over traditional imaging methods, which often lack the sensitivity to detect abnormalities at the earliest stages of the disease.

To further improve diagnostic accuracy, we constructed an integrated clinical-radiomics model, which improved model performance and reflects the multifaceted nature of PD. PD is a complex disorder with both motor and non-motor symptoms, and its diagnosis cannot rely solely on the presence of motor dysfunction (Kalia and Lang, 2015). Cognitive impairments, sleep disturbances, and autonomic dysfunction are common non-motor symptoms that significantly contribute to disease progression and patient morbidity (Leroi et al., 2012). The use of established clinical scales, such as the UPDRS, Hoehn-Yahr stage, and MMSE provided valuable insights into the severity and stage of the disease, especially when combined with imaging features, enhanced the model’s sensitivity and specificity. Our clinical-radiomics integrated model achieved an AUC of 0.983 in the training set and 0.837 in the test set, demonstrating a significant improvement in diagnostic accuracy compared to radiomics models alone. This aligns with previous research highlighting the benefits of combining clinical data with radiomics features for a more comprehensive diagnostic approach (Chougar et al., 2021). The integration of clinical variables not only enhances the model’s performance but also makes it clinically applicable, as it leverages existing clinical assessments commonly used in routine practice. This approach could be particularly valuable for clinicians who are looking for objective, quantitative measures to support their diagnostic decisions.

In addition, the integrated clinical-radiomics model demonstrated strong performance in the training set, with an AUC of 0.983. However, a noticeable performance drop to an AUC of 0.837 was observed in the test set, highlighting a potential discrepancy in the model’s generalizability. Several factors may likely attribute to this, including overfitting, differences in the data distributions between the training and test sets, and the inherent limitations of the test set itself (Woldaregay et al., 2019). In machine learning, the purpose of training any machine is to be able to get better predictions of the testing values by enabling the machine to generalize from the training set of all possible inputs (Dwyer et al., 2018). However, the challenge of overfitting is inherent due to the high variability present in most machine learning algorithms (Woldaregay et al., 2019). Overfitting can often be mitigated by using techniques such as regularization, using a separate validation set to evaluate the model’s ability to generalize (Akhter et al., 2024). Feature selection plays a crucial role in improving both accuracy and generalizability by selecting the most relevant variables (Dwyer et al., 2018). In this study, embedded feature selection was achieved using LASSO regression, which employed mathematical regularization to reduce the relative contribution of specific features to zero, thus effectively removing their influence and leaving the most predictive and nonredundant features. Furthermore, SVM was assessed for each feature, and the best features were chosen according to a predefined rule (Dwyer et al., 2018). The optimal feature subset was constructed using ten-fold cross-validation in our study, with the main purpose of evaluating the generalization power of the algorithm and comparing a set of multiple algorithms to find the best algorithm. The use of cross-validation helps to avoid overfitting by ensuring that the model is tested on data it has not seen during training, thereby providing a more realistic estimate of its performance on unseen data (Akhter et al., 2024). The strength of preprocessing within a cross-validated pipeline is that it allows for automatic optimization of statistical parameters for each step, balancing the importance of achieving maximum accuracy during training with the model’s ability to generalize to the test data. Despite these procedures were conducted to reduce overfitting and increase generalizability, the observed performance discrepancy between the training and test sets may still arise from differences in data distributions—such as variations in imaging protocols, sample characteristics, or even inherent differences between the data of training and test sets. These shifts in data distribution could reduce the model’s ability to generalize to new samples, leading to the observed drop in test set performance. The datasets should have a sufficiently large sample size to minimize the risk of overfitting. Future research should validate our findings in larger, multicenter cohorts to assess the robustness and generalizability of the clinical-radiomics integrated model across diverse populations and further confirm its clinical utility.

One of the most crucial aspects of our study was the validation of the models using an independent test set derived from the PPMI database. The test set, which was acquired using different MRI scanners and imaging protocols, provided an opportunity to assess the generalizability of the models. Despite these differences, our models performed well, demonstrating AUC values of 0.852 in the test set, which is comparable to other studies in the field (Sun et al., 2024). This highlights the robustness and potential clinical applicability of the proposed clinical-radiomics model. Second, our findings supported the use of advanced radiomics techniques in routine clinical settings. Given the relatively high diagnostic accuracy of the MRI-based models, radiomics could be employed as a supplementary tool, providing additional information to guide decision-making in clinical settings. As the technology becomes more accessible and automated, radiomics could play a central role in the standard diagnostic workflow for PD. Furthermore, DCA conducted as part of our evaluation showed that the clinical-radiomics combined model provides a substantial clinical benefit across a wide range of decision thresholds. DCA is a novel method for evaluating the clinical utility of diagnostic models, and its application in this study emphasizes the practical relevance of our model in real-world clinics.

This study has several limitations. Firstly, the sample size, although adequate for preliminary validation, is relatively small, particularly in the test set. Future studies should aim to validate our findings using larger, multicenter cohorts to assess the robustness and generalizability of the integrated model across diverse populations and clinical settings. Secondly, while T2-Flair and T1WI are commonly used in routine clinical settings, other advanced imaging modalities, such as diffusion tensor imaging (DTI), functional MRI (fMRI), and iron-sensitive imaging, could provide complementary information and enhance the model’s diagnostic accuracy. Another limitation is the cross-sectional nature of our study. Longitudinal data would provide a more robust assessment of how radiomic features evolve over time and their ability to track disease progression. Future studies should focus on using radiomics to predict long-term outcomes, such as cognitive decline or motor progression, which are key concerns for clinicians managing PD patients. Additionally, while our integrated model combined clinical and imaging data, further research is needed to identify additional biomarkers that may improve diagnostic performance. Genetic, biochemical, and other neuroimaging markers could be incorporated into future models to capture the full spectrum of pathological changes associated with PD.

## Conclusion

In conclusion, this study developed an optimal radiomics signature in combination with clinical data model to markedly improve the diagnostic accuracy of PD, outperforming models that relied solely on imaging or clinical features. The integration of structural imaging biomarkers with clinical indexes not only improves diagnostic accuracy but also provides a deeper understanding of the neurobiological changes underlying PD. This model could serve as a non-invasive, easily accessible tool which, in conjunction with clinical variables, aiding in the early detection of PD and facilitating timely interventions. Moving forward, longitudinal studies should concentrate on validating a multimodal combination of structural imaging techniques, functional images, and potentially other modalities (e.g., inflammation, other neurotransmitters) in larger, more diverse populations with the aim of improving patient outcomes through earlier and more accurate diagnosis and providing stronger interpretation of disease-modifying therapies.

## Data Availability

The raw data supporting the conclusions of this article will be made available by the authors, without undue reservation.

## Footnotes

1
http://www.ppmi-info.org

2
https://www.ppmi-info.org/study-design

3
http://www.Rprojiect.org
